# Clinical Society of St. James Dispensary

**Published:** 1898-02

**Authors:** 


### *SOCIETY REPORTS.*

### CLINICAL SOCIETY OF ST. JAMES DISPENSARY.

SAVANNAH, GA., January 1, 1898.

The regular paper of the evening on "Quinine in Malaria, Excluding the Simple Intermittents," was read by Dr. Van Marter. (Published in this number.)

DISCUSSION.

*Dr. Graham*: I have found quinine in the *continued form of malarial fever* to be anything but a specific. Lauder Brunton brought out the fact that when bile is present in the stomach quinine by the mouth does little or no good, as it is rendered insoluble and is not absorbed.

It is my opinion that in this disease we have to deal with a toxemia, perhaps the toxin produced by the metabolism of the parasite of malaria, or more likely the lowered resistance and the altered condition of the blood and intestinal tract engendered by these parasites favors the bacillus coli communis in assuming a pathogenic form, and we really have, as Dr. Woodward long ago named it, typho-malarial fever. The bacillus coli communis, ordinarily an innocent saprophyte, may, under other conditions, perhaps, become pathogenic and a very near relative to the typhoid bacillus. I do not mean to say that we have true typhoid and malarial fever at the same time in the same subject, but that after the plasmodia are no longer to be found in the blood by the microscope, the fever continues unabated and unaffected by quinine; therefore it is evident to me that the fever is either due to malaria toxin, or, more likely, to a toxin elaborated by the bacillus coli communis, over which quinine has no beneficial influence or effect.

Quinine has been very disappointing to me in this disease, and I have found it of little or no value unless supplemented by other drugs. Warburg's tincture, either the liquid or powdered extract, has served me well. There are cases that die with quinine given, even in heroic doses, at the proper time and despite all we can do.

*Dr. Lanier*: Dr. Van Marter's paper is on a line of observation that means a great deal to us as practitioners in this malarial region. I fully agree with the writer in that we do not have in quinine the best treatment for some forms of malarial fever, nor even in some of the irregular forms of intermittent.

I feel safe in saying that nearly every physician in our Southern country who is familiar with what was so long termed bilious remittent fever will agree with Dr. Van Marter about the necessity for giving other drugs than quinine in continued malarial fever. And my opinion is, that if we have administered quinine freely for four or five days without getting well-marked morning remission, with moist tongue and skin, we had better leave off quinine and use some other remedy. If we would study the use of the various preparations of quinine, I believe we should find some form suited to nearly all conditions of the stomach, for it is a fact that the plain sulphate of quinine in the stomach of a patient who is nauseated, and perhaps vomiting mucus and bile, is only an increasing irritant. My experience is that in nearly all malarial conditions we get a better effect from the bisulphate than from either the sulphate or the hydrochlorate. I mention these preparations because it is upon one of them that we rely in nearly all cases where quinine is used, except in the use of the drug hypodermically.

Quinine in suitable doses, at the proper time, seems to be a specific for malarial affection in those cases which have the nearest approach to marked intermittency; therefore, the more continuous the symptoms the less does it act as a specific.

Quinine certainly does not cure every case of malarial fever, even though you have the patient *thoroughly cinchonized.* I have seen patients fully deafened by quinine and the fever go along unabated. And it is here that we do our patient an injury with the drug. Malarial fevers of nearly all types *can be cured without quinine!* Yet we have become so wedded to the remedy that we are inclined to discount other drugs, and forget the fact that it is not very uncommon to find persons with malarial fever who cannot take quinine from some existing idiosyncrasy. However, it is our good fortune to pull these patients through all right. About three years ago I saw a man who had a typical malarial fever, who contracted his sickness while digging up the earth for laying gas-pipes; his fever was markedly remittent. Of course, I thought of quinine at once as the remedy, and before I finished writing a prescription the patient assured me that he could not take quinine in any form; but I unwisely concluded that his previous medical attendants did not know as much about the administration of the drug as myself, and I forthwith ordered an acid solution of bisulph. quinia, thinking that I would get a quick effect, and that if any unpleasant symptoms should follow I would relieve them with bromide, antipyrin or morphine. In an astonishingly short time after the patient took the first dose I was called back to find him suffering from a terrifying rash, nauseated, nervous, greatly depressed and exceedingly bad off. After a few hours the quinine symptoms disappeared, but the fever rose to a high point. Again, I concluded that it was on account of the quinine in the stomach, irritating the mucous membrane, that had produced the bad symptoms; consequently, the following morning, I injected the drug hypodermically and administered it per rectum, for I was determined to give it a fair trial, particularly so because the malarial character of the attack was so well defined. Again, the second time, I was forced to regret my experiment, because my patient was no better, and secondly, because I had produced a new set of symptoms by having administered quinine. I immediately ordered three drops of apiol every two hours, alternating with a formula so much used by Dr. Corson of this city, which contains Fowler's solution, tr. nux and tr. gelsemium. It was only about forty-eight hours after beginning this course of treatment when my patient was relieved of all his neuralgic pains and the fever began to fall. From that time it was plain sailing, for the patient got rapidly well. If I had kept a record of cases it would be easy to show that, in a number of patients who were unable to take quinine I had pulled them through with other drugs.

*Dr. Oliveros*: I have heard it said here to-night that quinine can be given in very large doses, without getting any bad effects in the head, or otherwise. We have heard from one of the gentlemen present a very good definition of a specific. I now think that we are sadly in need of a definition of bad "effects." I have heard for the first time in my life, from one of the physicians here, that quinine is given in doses of 60 to 100 grains, repeated in one or two hours. Such a procedure I think absurd, and nothing more than a waste of the drug. If no bad effects are had, it is simply, as must necessarily be the case, that there is no absorption of that amount, or anything like it. If 5 or 10 grains of quinine repeated every two hours, for a certain length of time, will not produce the desired effect on the fever in some particular case, it is my practice and my opinion that the drug should be discontinued and some other plan of treatment adopted.

As I am here this evening only as an invited guest, I have not looked up the subject, but there are a great many points in Dr. Van Marter's paper with which I agree fully, and I think he has treated the subject in a very able manner. I must add my testimony to his, that, in the continued form of malarial fever, quinine has most decidedly little or no effect. In a great majority of cases it is a waste of time and quinine; in fact, I might add that, personally, I never use the drug in these cases with one exception, and that is when there is a very sharp remission, especially at the tail end of the case. I then give two ten (10) grain doses, with the hope that I may get result enough to finish the case; in other words, I give the patient the benefit of the doubt; but even then it is my experience that the physician gets more disappointment than the patient does benefit.

It is not only in the continued form of malarial fevers that quinine proves itself no specific. I have seen many cases of the intermittent form where the specific action seemed very slight. Any country physician will have run across cases where quinine has been given faithfully, in correct doses and at proper times without any benefit until that particular patient has been moved from the neighborhood in which he contracted it. Likewise in malarial cachexias quinine evidently is no specific, in fact, seems to do very little good without the usual auxiliary treatment.

*Dr. Fitch*: I have listened to Dr. Van Marter's paper and the discussions with much interest. Quinine is not the specific which northern clinicians claim it to be. They do not see malaria as it is found in its natural home. I have repeatedly treated cases of malarial poisoning in which quinine prescribed in any form, dosage, manner or time of administration, had no effect whatsoever in controlling the fever. My observation leads me to assert that quinine is not a specific or antidote to the plasmodium of the grave, continued malarias or the toxins produced by them. Yet in the milder tertian and quartan types of malaria quinine is a true specific if given in the proper dose and at the proper time.

I had under my care not long since a case of malaria (proven by microscopic examination), algid form, pernicious type, in which quinine had been given in small doses for weeks as a prophylactic, yet this did not prevent the development of malaria. I prescribed large doses; 20 grains bisulphate of quinine, hypodermically, every four to six hours, produced cinchonism, yet the fever continued, with regular paroxysms daily. I then gave the same preparation dissolved in normal saline solution subcutaneously, and later by intravenous injection, none of which influenced the disease. I saw this patient die, and witnessed the failure of quinine as a specific.

I have had only a limited experience with quinine in malarial hemoglobinuria. I, in common with many others, have come to the conclusion that hemoglobinuria, if not actually caused by quinine, is a condition in which quinine is an absolute poison. It is claimed by many writers (Plehn, Richardson and others) who have had favorable opportunities to study the action of quinine in these cases, that quinine may produce these grave symptoms, and that it is of no value as a remedy in these cases.

*Dr. Corson*: I differ greatly with the views expressed by Dr. Van Marter. In a paper of this character the doctor should have defined in the beginning what he meant by a specific, for, taking malaria in its entirety, quinine is a specific and is so recognized all over the world. By a specific we mean that drug or substance which has the special property of preventing or curing a particular disease. Of course, taken in its absolute sense, no such remedy exists, for there is no specific that will prevent or cure *every* case of that particular disease over which it has this reputed special influence. Even those drugs most worthy of the name fall far short of being absolute specifics; though they may cure a large proportion of the cases brought under their influence, a certain proportion will go free.

The continued type of malarial fever means a greater involvement of the system from the malarial poison than in the simple intermittent type. That is recognized, and it stands to reason that with this greater poisoning the less rapid and the less effective is the action of quinine. And we also see cases where this poisoning has gone so far that neither quinine nor any other drug avails, and the patient will die. I know that there are other drugs than quinine which will cure intermittent fevers. I know that in certain forms of malarial toxemia and anemia quinine alone is quite powerless to effect a cure, and thatiron, arsenic and strychnia, with the mineral or organic acids, must be brought in. I know that in certain individuals quinine greatly aggravates the case, due to a peculiar idiosyncrasy; and I have even seen cases where quinine might act as a fatal poison if given in large doses; and yet I will state most emphatically that after treating malarial affections in Savannah for eighteen years, I have cured by quinine a large majority of my cases of continued remittent fever, of larval forms of malarial fever, due to mild yet chronic malarial toxemia, and a fair proportion of these chronic forms when the toxic symptoms are more marked. The fact that the mortality is high in the pernicious malarias, be they hemorrhagic, icteric or algid in type, does not militate against the specific action of quinine. When the malarial toxemia has gone this far there is such a paralysis of nerve centers that the system responds not at all, or but slightly to any drug.

In certain continued types of fever in Savannah, which are plainly not malarial, but point to an enteric intoxication, be it a true typhoid, or due to the bacillus coli communis, or to other toxic agents from the alimentary canal, I admit that quinine is useless; in fact, I know of no drug which cuts the disease short. In the beginning, however, before the nature of the fever is determined, I give quinine to prove or disprove the malarial character of the case. I regard this therapeutic test as a valuable one. I thus go so far as to state that, in my opinion, quinine is most worthy of the name of specific in malaria, be it intermittent or remittent in type; that it is always the first drug to be considered, and that it is only when conditions arise which are *secondary* to the acute primary poisoning, that we must call in the aid of other drugs.

*Dr. Daniel*: After hearing Dr. Van Marter read his paper, I am inclined to think the doctor rather exacting in his ideas as to what constitutes a specific. As for quinine being a specific for all the sequelæ and complications of malaria, no one has ever set forth such claims, any more so than that antitoxin and mercury will correct all the sequelæ of diphtheria and syphilis, and yet they are recognized as specifics if administered at the proper time and in right amounts, depending upon the stage of case and patient.

So we must bear in mind in discussing this paper that there is no such thing as a specific in the narrow sense of the word, but as ordinarily used, quinine is the *only specific* for malaria. In the majority of instances the failure to recover on the part of the patient is not due to the want of anti-malarial properties in the quinine, but to the timidity of the physician, who fears a harmless drug, and as a result will attempt to cure a disease that has permeated to the most remote parts of the human body and is sapping one's life-blood with the pitiful amount of 3 to 15 grains of quinine, administered in twenty-four hours, and here they will stop and say the patient is cinchonized, because he has "tinnitus aurium," and quinine given above this amount will pass through the body unchanged and have no effect. Let me ask the advocates of this theory —for it is nothing but a theory—if they would stop instilling atropia into the eye for an iritis with the pupil to a half or three-fourths dilatation and some dryness of the throat? There are your physiological effects, but you dare not stop the atropia until your iritis subsides and you carry it to a maximum dilatation of the pupil if needed to accomplish the desired end. So I will beg of the gentlemen to remember that the dose of any drug is not the amount it takes to have a phyiological effect, nor is it a fixed amount, but is the quantity that it takes to have the desired results in the particular case under consideration. So in administering quinine, don't stop with 5 grains a day because the ears ring, but give the amount that it takes to break up the fever and prevent a return of the malarial paroxysm; give 30, 90 or 120 grains, if needed, or even more, and you will surely cure your patient, unless he was moribund when you called to see him.

Another important factor in administering quinine is the form in which it is given. I prefer the sulphate and it either in solution with dil. sulphuric acid or made up in fresh pills with dil. sulphuric acid and glycerin. Again, I must differ with the doctor, in that I prefer giving quinine in the stomach, when possible. My limited experience has about convinced me that subcutaneous injections of quinine for the cure of malaria are about on a par with the subcutaneous injections of bichloride of mercury for the cure of syphilis, which is another good theory. We can get all of the physiological effects of the mercury as well as of the quinine, but we all know that the injections of mercury will not cure syphilis in as short a time as is claimed, nor have I found quinine given under the skin to act nearly so efficiently as when given in the stomach.

Dr. Van Marter states that very often in the "hemoglobinuric" form of malaria, quinine will cause a return of the so-called bloody urine. Again I must differ with the doctor, and would like to ask him if *he* ever saw a case in which it occurred. I have seen a few cases of hemoglobinuria due to malaria, and in the intermittent type I have never seen a recurrence of the hemoglobin after administering quinine in doses of 30 to 60 grains six hours prior to the anticipated paroxysms. If you have a remittent type of malarial hemoglobinuria, don't wait for the fever to go off (for it might be the patient instead of the fever that will go off), as I have heard advised, for it is not going off unless you make it with large doses of quinine. And begin giving it immediately you see the patient; give him from 60 to 120 grains within an hour. If his stomach will not tolerate it, put it under his skin as next best. And I must say that I think Dr. Van Marter's idea of diluting the solution well is a good one. But, gentlemen, never be afraid of quinine causing a recurrence or increase of the hemoglobin; it is want of quinine that does it. As for malarial hematuria, I have never seen it unless as a local trouble in connection with a cystitis.

